# Effects of Purple Seed Stain on Seed Quality and Composition in Soybean

**DOI:** 10.3390/plants9080993

**Published:** 2020-08-05

**Authors:** Richard E. Turner, M. Wayne Ebelhar, Teresa Wilkerson, Nacer Bellaloui, Bobby R. Golden, J. Trenton Irby, Steve Martin

**Affiliations:** 1 Delta Research Extension Center, Mississippi State University, 82 Stoneville Road, Stoneville, MS 38776, USA; webelhar@drec.msstate.edu (M.W.E.); Twilkerson@drec.msstate.edu (T.W.); Bgolden@drec.msstate.edu (B.R.G.); steve.martin@msstate.edu (S.M.); 2 USDA-ARS, Crop Genetics Research Unit, 141 Experiment Station Road, Stoneville, MS 38776, USA; nacer.bellaloui@usda.gov; 3 Department of Plant and Soil Sciences, Mississippi State University, 32 Creelman Street, Starkville, MS 39762, USA; tirby@pss.msstate.edu

**Keywords:** soybean, seed composition, seed protein, seed oil, seed health, purple seed stain, *Cercospora kikuchii*

## Abstract

Purple seed stain disease, caused by (*Cercospora kukuchii*), is a major concern in soybean (*Glycine max* (L.)) in Mississippi, USA, due to its effects on seed quality, reducing soybean seed grade and potential market price at elevators. Therefore, investigating the effects of purple seed stain (PSS) on seed quality (germination and vigor) and seed composition (nutrition) is critical. The objective of this study was to investigate the effects of PSS on seed harvest index, seed germination, seed vigor, and seed composition components (protein, oil, fatty acids, and sugars). A field experiment was initiated in 2019 in Stoneville, MS, at the Delta Research and Extension Center (DREC) on a Commerce silt loam soil (fine-silty, mixed, superactive, nonacid, thermic Fluventic Epiaquepts). Soybean variety Credenz 4748 LL was used. The results showed that infected (symptomatic) seed had a 5.5% greater Seed Index (based on 100 seed weight) when compared to non-infected (non-symptomatic, as control) seed. Non-infected seed had greater percent germination and seedling vigor when compared to infected seed. Germination was 30.9% greater and vigor was 58.3% greater in non-infected seed. Also, the results showed that infected seed with PSS had higher protein content and some amino acids. No changes in total oil and fatty acids. Sucrose and stachyose were lower in infected seed than in non-infected seed. The research showed that PSS impacted seed health and seed quality (germination and vigor) and seed composition (protein, sugars, and some amino acids). Purple stained seed should be avoided when planting and should be managed properly as low germination is a potential risk. Planting population should be adjusted accordingly due to lack of germination and vigor if PSS is present. This research help growers for purple seed management, and scientists to further understand the potential negative impact on seed quality and nutrition. Further research is needed before conclusive recommendations are made.

## 1. Introduction

Purple Seed Stain (Cercospora blight), caused by the fungus *Cercospora kikuchii,* is a major disease in Mississippi, USA, and also a worldwide disease, leading to purple seed [1], causing reduced market grade, poor processing qualities, and reduced seed vigor [2,3]. Soybean seed quality and composition are major component determining the seed health and seed nutritional value. Seed quality, generally, refers to germination and vigor; seed composition refers to seed protein, oil, fatty acids, amino acids, sugars, and other constituents such as isoflavones and phenolics [4,5]. Seed quality and composition can be influenced by several factors, including abiotic (for example, heat, drought, and soil conditions) and biotic (for example, genotype and diseases/pathogens) factors [4,5], resulting in poor seed quality and reduced seed nutritional qualities. Major components that influence seed quality are seed germination, vigor, nutrients content, and seed health. Secondary factors that influence seed quality are seed size, seed density, mechanical damage, and seed moisture content [6]. Since information on the effect of PSS on seed quality and composition is limited, the current research will focus on the effects of purple seed stain (PSS), caused by *Cercospora kukichii*, on seed quality (germination and vigor) and seed composition (protein, oil, fatty acids, amino acids, and sugars). Standard seed germination refers to the performance of seed under desirable and favorable planting and environmental conditions. Seed vigor refers to the potential performance of seed under adverse planting and environmental conditions by estimating the seedling growth and health over time using growth test. This test is designed to indicate that seeds with high vigor grow at a faster rate as compared to seeds with low vigor potential. The differences in seedling growth rate can be observed to indicate seed vigor. Generally seed vigor is indicated by metabolizing food reserve more faster than low vigor seed, leading to faster germination, and a strong establishment of stand and seedling in the field compared with those with poor vigor. Therefore, quick growth rate of seedling indicates stronger seed vigor.

Although seed quality and composition are genetically controlled, environmental factors, including drought, heat, and diseases showed to significantly affect the quality components of the seed. For example, conditions, such as delayed planting, have been documented to increase seed linolenic acid and protein levels and reduce oleic acid and oil levels [7]. However, delayed planting had little to no effect on linoleic, stearic, and palmitic acid. They concluded that year had a larger impact on seed composition than planting date, and that certain acids were dependent upon maturity group and temperatures during grain-fill [7]. A comparison of seed composition of two soybean cultivars under both irrigated (full season and reproductive), and rain-fed conditions [4]. Irrigation resulted in significantly greater soybean grain yield in five of six site years when compared to no irrigation. Full season irrigation produced greater soybean grain yield when compared to no irrigation in six of six site years. A large increase in commodity price would be required for specific seed composition in order to justify growing non-irrigated soybean if irrigation is possible [4]. Research on soybean under drought conditions (control, moderate, and severe) and observed the impact of temperature (28 and 34 °C) effect on seed composition [8]. They suggested that drought can increase seed protein by up to 11.8% and reduce oil by up to 12.4%. Similarly, [9] found the greatest seed oil was achieved under well-watered conditions. High air temperature averaged across years reduced seed protein by 29%. Results concluded that air temperature and drought have an impact on seed quality, quantity, and seed size proportion. An inverse relationship was shown with temperature and seed oil content [10]. Environmental conditions that increase protein such as high temperatures and drought all significantly reduce soybean grain yield. 

Previous research on disease effects such as charcoal rot, Phomopsis, and purple seed stain on seed quality and composition was reported, but was not completely conclusive. For example, when soybean seed germination was compared between infected seed with PSS and noninfected seed [11,12,13]. It was found that the reduced germination rate was associated with infected soybean with additional infection of other pathogens such as *Phomopsis* spp. and not the *C. kukuchii* [12]. Research by others [13] determined that *C. kukuchii* infection did not increase with delayed harvest; however, infection caused by *C. kukuchii* was greater than (*Diaporthe or Alternaria*) on both protected (protected using pollinating bags) and nonprotected soybean pods [13]. 

Roy [14] analyzed the infection cycle of *C. kukuchii* on soybean. Research conducted by [15] concluded that healthy plants on ends of rows can become infected; therefore, infection is not due to weakened plants. Differences in length of flowering, due to different maturity groups, and flowers (opened, unopened and open for multiple day) did not increase infection [11,14,16] concluded that *C. kukuchii* infects developing seed pods, and suggested that pod age at time of infection could influence amount of infected seed. Reference [14] observed the majority of infected soybean pods were located on the main stem and top half of the plant. Reference [17] concluded that the percentage of purple seed stain on the seed coat (severity) of infection has an influence on seed germination. On the other hand, plant stress caused by stinkbug feeding has also been shown to alter soybean seed composition [18,19,20], and was found that insect feeding increased total seed protein level when compared to nondamaged soybean seed [18,19]. Since total seed protein and total oil are inversely correlated, increasing total seed protein levels, total seed oil was reduced when compared to the nondamaged soybean seed. Levels of linoleic, palmitic, stearic, and oleic fatty acids all increased, but linolenic fatty acid content decreased as a result of stink bug damage [20]. 

Based on the above discussion, it was shown that the effects of seed diseases, especially PSS infection on seed quality and composition is a major concern in Mississippi and its investigation is critical. Therefore, the objective of this research was to investigate the effects of PSS on seed harvest index, seed germination and vigor, and seed protein, oil, fatty acids, amino acids, and sugars. 

## 2. Result

### 2.1. Soybean Seed Index, Seed Germination, and Seed Vigor

**Figure 1:**
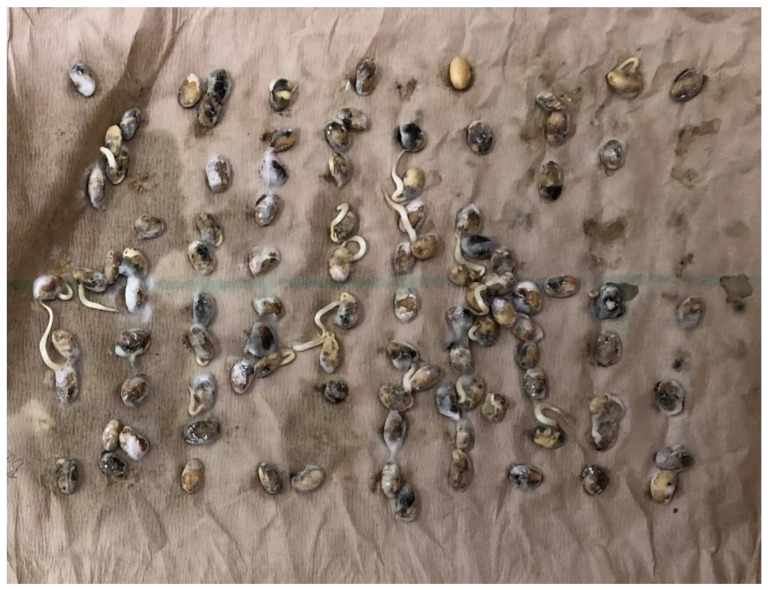
Seven days after initiation of germination and seedling vigor test. Incubators set at 25 °C on continuous light. An image representing seedling germination and vigor of infected soybean seedlings. The field experiment was conducted in 2019 at the Delta Research and Extension Center in Stoneville, MS.

**Figure 2:**
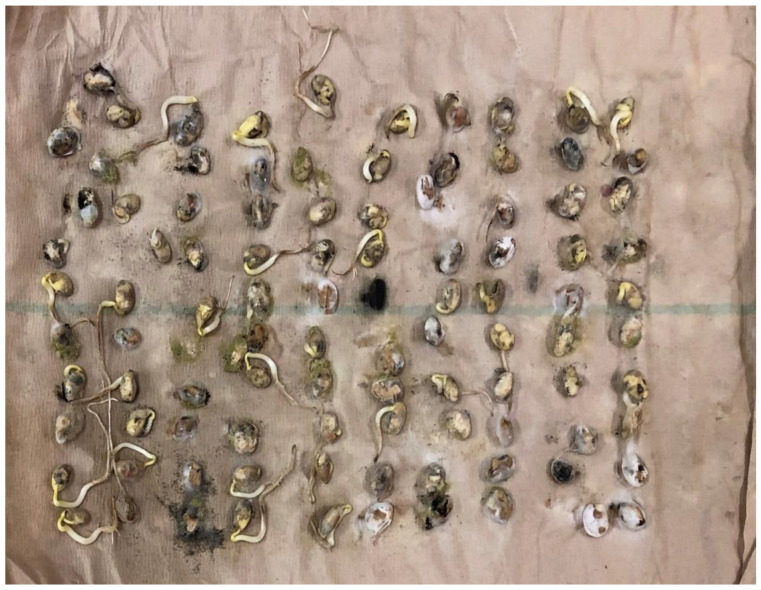
Seven days after initiation of germination and seedling vigor test. Incubators set at 25 °C on continuous light. An image representing seedling germination and vigor of non-infected (non-symptomatic) soybean seedlings. The field experiment was conducted in 2019 at the Delta Research and Extension Center in Stoneville, MS.

Seed Index was 5.5% greater when comparing infected soybean seed to non-infected soybean seed (Table 1). Several stress factors such as drought and insect feeding have been reported to reduce Seed Index [9,19]. Data from this analysis suggested that infected soybean was better quality than non-infected soybean seed. Non-infected seed had an increase of 58.3% of seedling vigor and an increase of 30.9% of seed germination when compared to infected soybean seed (Table 1). Reference [21] reported that PSS caused by *C. kikuchii* did not reduce germination; however, seed in this study were separated by visual symptoms of PSS. Plants may have been infected with the disease but not show symptoms of PSS (Figure 1 and Figure 2) show the difference in seedling germination and vigor. Data shows the importance of clean/healthy seed if growing seed soybean for planting. Data should be used to standardize seed selection to prevent lowering germination on individual bags of seed. 

### 2.2. Soybean Seed Composition

The non-infected soybean seed had greater sucrose and stachyose when compared to infected seed (Table 2). Both sucrose and stachyose are main sugars in soybean that contribute to total carbohydrates along with raffinose. Non-infected seed showed an increase of 26.3% greater sucrose when compared to infected soybean seed. Non-infected seed had 10.8% greater stachyose than infected. No changes in total oil and raffinose were observed. PSS resulted in changes in total protein and several amino acids (Table 3 and Table 4). Infected soybean seed had significant increases in 7 out 18 amino acids, including total seed protein, which was increased by 2.6% when compared to non-infected seed. Non-infected seed when compared to infected seed resulted in similar cysteine seed composition (NS). No differences were observed between infected and non-infected for 10 out of 18 amino acids (Table 3 and Table 4). Previous research showed that soybean plants that were exposed to stress produced seed that were greater in total protein when compared to soybean seed that were not [4,8,10,22]. Neither soybean seed type produced significantly greater fatty acid seed (Table 5). Unlike typical plant stress which causes alteration to fatty acid composition within the infected soybean seed compared to non-infected seed did not.

## 3. Discussion

The higher harvest index in infected than in non-infected seed may indicate that the PSS did not reduce yield, but rather PSS can increase the yield, especially PSS antagonizes other fungal diseases such as Phomopsis and charcoal rot, and others. The lower germination rate and vigor in infected seed compared to non-infected seed were previously reported. Previous reported indicated that the effect of PSS on germination is unclear [23]; however, [2,24] showed that seedling emergence from infected seed with PSS was reduced by 7–15% under greenhouse and field conditions, bearing in mind that environmental conditions are important to the development and severity of PSS [25]. Since resistances to Cercospora and Phomopsis seed infection were found to be controlled by single dominant genes [26], and negative correlations between incidences of seed infection by *C. kikuchii* and Phomopsis spp. have shown to have an antagonistic interaction, Cercospora infection inhibits infection by Phomopsis spp., especially in soybean genotypes that are very susceptible to Cercospora seed infection [23]. Other reports, however, indicated that, using F2 populations from AP3503 PI80837 and PI911133 PI 80837 (N 5 222 plants), the negative correlation was not significant [3,27]. The lower sucrose and stachyose, and higher protein, and alteration of some amino acids in infected seed than in the non-infected seed were shown by previous researchers, although the effects of fungal diseases, including PSS is still not well understood, and what is available in the literature is still conflicting. For example, it was reported that high percentage of infected seed increase protein content and decrease fatty acids [28]. Reference [29] reported that seed infected by PSS resulted in lowed fatty acids stearic, palmitic, linoleic, linolenic, and protein, but higher oleic and total oil. Other research, working on other fungal diseases such as charcoal rot, found that infested seed with charcoal resulted in lower seed protein and linolenic acid, but higher in oleic acid [4]. Others, working on Phomopsis fungal disease found lower protein and oil concentrations were observed in seed susceptible to Phomopsis compared with other resistant or moderately resistant [5]. This was explained as that the susceptible seed to Phomopsis has weak cell wall or weak seed coat integrity compared with moderately resistant or resistant genotypes. Weak cell wall or lack of seed coat integrity may lead to loss of soluble compounds from seed or lead to pathogen penetration through the seed coat, resulting in breakdown of organic compounds, including protein and oils and may lead to leakage and loss of soluble compounds [30]. Soybean seed protein and oil are rich in essential amino acids and fatty acids, which are favorable conditions for fungal pathogens to grow and multiply [31], leading to chemical breakdown of protein, oil, and fatty acids [32]. 

Therefore, conflicting results still exist regarding the effects of fungal diseases on seed composition. Reports on Phomopsis fungal disease ranged from no effects on seed protein and oil; to significant negative correlation between seed infection and seed palmitic and oleic acids, to a significant positive correlation between seed infection and linoleic and linolenic acids [33]. Others reported higher oil and protein percentages in seed with symptoms of *Phomopsis sojae* infection [34]; a positive correlation between soybean seed protein concentration and fungal damage by *Fusarium* spp., *Cercospora* spp., and *Phomopsis* spp. [35]; and higher seed protein in seed inoculated with *Colletotrichum truncatum* than those in the un-inoculated seeds [32]. It was also reported that fungal-infected soybean seed showed higher protein concentrations [36], lower carbohydrates, and no change or increased oil concentrations [23]. 

Our research showed that infected seed with PSS showed reduction in germination and vigor, agreeing with those of [23,24]. Infected seed showed superiority in protein and some amino acids agreeing with those of [23,32,34,35,36]. Also, our research showed a decrease in sucrose and stachyose, and no changes in oil, fatty acids, disagreeing with those of previous research who noticed a reduction in palmitic, stearic, linoleic and linolenic [28,29].

## 4. Materials and Methods

### 4.1. Field Conditions and Seed Collections

The experiment was conducted at the Mississippi State University Delta Research and Extension Center near Stoneville, MS, USA. A field trial was conducted on silt-loam soil composed mainly of a Commerce silt loam that is commonly cropped to soybean within Mississippi. Trial evaluated ‘Credenz 4748LL’ planted on 102-cm beds using plots that were 30.5-m in length and 4.1-m in width. Soybean was panted on 25 April, 2019, and field was exposed to routine alternating management strategies (fungicide, desiccant, additional P and K fertility, and additional N and S fertility). Mature seeds were collected to conduct seed composition analysis, seed index, vigor, and germination of non-infected (non-symptomatic seed) and seed infected with purple seed stain (PSS). Soybean seed was then sorted into non-infected (non-symptomatic seed) and seed with symptoms of PSS. Both seed types had the seed coating removed by splitting each seed. A 100 soybeans were collected randomly to evaluate Seed Index. A 100 seeds of each seed type were used to evaluate seed germination and seedling vigor. Germination was determined by counting the number of germinated seed after seven days (temperature) 25 °C. Seedling vigor was determined on a scale of 1 to 9, with 1 being weak vigor and 9 being the most robust. 

### 4.2. Measurements of Seed Quality Components

Non-infected and infected seed, once shelled, were ground using a Laboratory Mill 3600. Seed samples of 25 g were used to determine seed analysis for protein, oil, and fatty acids using NIR, with a diode array feed analyzer AD 7200 (Perten, Springfield, IL USA) [37]. An initial calibration equation was developed at University of Minnesota using Preteen’s Thermo Galactic Grams PLS IQ software. 

A 100-seed sample from each seed type was collected for Seed Index. Samples were weighted using a calibrated Denver Instrument Company XE series model 400 balance (Denver Instrument Company, 5 Orville Dr.; Bohemia, NY, USA). Each subsample was subjected to a seed germination test. Briefly, (i) wetting germination paper with distilled water (ii) placing 100 seed in middle of paper using a fill tray (iii) cover seed with second sheet of wet paper (iv) roll tightly and secure with rubber band on each end (v) seed were placed upright in a beaker and left to incubate at 25 °C for seven days (vi) seed were counted for each rep that had germinated. Seed vigor was estimated by using 100 seeds and visually observed the seedling growth and count them. We used a scoring of 1 to 9, with 1 being the poorest (0%, 0 seed germination) and 9 being (100%, all 100 seeds germinated). Same starting procedure was followed to asses seed vigor, but after seed germination, the 100 seeds were observed for further growth rate for root, shoot, and size, and visually scored from 1–9, with 1 being the poorest (0 % of 100 seed) and 9 being the highest (100% od 100 seed). So, the seedling growth rate was a visual rating based on root and shoot growth and size, and visually scored from 1 to 9, as explained above, and the bigger more robust sprouts received a greater vigor rating. 

### 4.3. Experimental Design and Statistical Analyses

The study was setup with a completely randomized bloc design with four replicates. Mature seeds were collected as previously described and Seed index, seed germination, seed vigor, and seed composition were determined. Data were subjected to ANOVA using the PROC MIXED procedure in SAS v. 9.4 (SAS Institute Inc., 100 SAS Campus Drive, Cary, NC, USA) [38]. Data were analyzed using Type III statistics in SAS (SAS v. 9.4 (SAS Institute Inc., 100 SAS Campus Drive, Cary, NC, USA) to test for treatment effect (infected compared with non-infected), and least square means were separated at the *p* ≤ 0.05 significance level. Least square means were calculated and mean separation (*p* ≤ 0.05) was produced using PDMIX800 in SAS, a macro for converting mean separation output to letter groupings if a significant response occurred [39].

## 5. Conclusions

Infected seed had increase Seed Index which could result in more bushel when being marketed, confirming that PSS does not affect seed yield. Typically, infected seed is viewed as problematic but data from this research suggest PSS infected seed is superior in some seed composition components. Decreased levels of sucrose in purple seed stained seed could increase survivability of the disease on those seeds. However, the pathogen does show decreased seedling vigor and germination in the study. If soybean seed was to be crushed and used for protein and oil content, then producers should not be penalized for seed that is infected with PSS. However, if the seed were designed for planting soybean seed (seed used for production) then the presence of PSS does lower germination and vigor. This data indicates that PSS infection is likely occurring late enough in the growing season to not harm most of seed composition constituents. However, the presence of PSS does have carryover effect (lower vigor and germination) to the next growing season if infected seed were to be planted. 

## Figures and Tables

**Table 1:** The comparison of non-infected (non-symptomatic) compared to infected soybean seed for Seed Index, vigor, and germination. The field experiment was conducted in 2019 at the Delta Research and Extension Center in Stoneville, MS.

Component	Non-Infected ^x^	Infected ^y^	LSD	Pr > F
Seed Index (g 100 seed)	16.4200 ^b^	17.3800 ^a^	0.4424	0.0010
Vigor (%)	6.0 ^a^	2.5 b	1.2	0.0004
Germination (%)	59.3 ^a^	41.0b	8.1	0.0015

Means within a row followed by different letters are significantly different at *p* ≤ 0.05. (^x^ non-symptomatic seed showed no sign of purple seed stain (PSS); ^y^ infected seed had visual symptoms of PSS infection. Vigor rated on scale of 1–9 (1 being little growth and 9 being most growth).

**Table 2:** The comparison of non-infected (non-symptomatic) compared to infected soybean seed as it influenced seed total oil, sugars, and fiber content. The field experiment was conducted in 2019 at the Delta Research and Extension Center in Stoneville, MS.

Component	Non-Infected ^x^	Infected ^y^	LSD	Pr > F
	(%)			
Total Oil	28.2 ^a^	28.2 ^a^	-	0.8818
Sucrose	3.8 ^a^	2.8 ^b^	0.4	0.0003
Raffinose	0.8 ^a^	0.7 ^a^	-	0.2244
Stachyose	3.7 ^a^	3.3 ^b^	0.3	0.0446
Fiber	3.3 ^a^	3.2 ^a^	-	0.6393

Means within a row followed by different letters are significantly different at *p* ≤ 0.05. ^x^ non-symptomatic seed showed no sign of purple seed stain (PSS); ^y^ infected seed had visual symptoms of PSS infection.

**Table 3:** The comparison of non-infected (non-symptomatic) compared to infected soybean seed for seed total protein and amino acids content. The field experiment was conducted in 2019 at the Delta Research and Extension Center in Stoneville, MS.

Component	Non-Infected ^x^	Infected ^y^	LSD	Pr > F
	(%)			
Total Protein	36.8 ^b^	37.8 ^a^	0.7	0.0093
Aspartic Acid	4.2 ^b^	4.3 ^a^	0.1	0.0233
Threonine	1.8 ^a^	1.8 ^a^	-	0.6337
Serine	1.7 ^b^	1.9 ^a^	0.1	0.0239
Glutamic Acid	4.8 ^a^	4.9 ^a^	-	0.6645
Typtophan	0.5 ^a^	0.5 ^a^	-	1.000
Proline	2.1 ^b^	2.3 ^a^	0.1	0.0004
Glycine	2.2 ^a^	2.3 ^a^	-	0.1295
Alanine	1.8 ^b^	1.9 ^a^	0.1	0.0172
Cysteine	0.5 ^a^	0.4 ^a^	-	0.2462

Means within a row followed by different letters are significantly different at *p* ≤ 0.05. ^x^ non-symptomatic seed showed no sign of purple seed stain (PSS); ^y^ infected seed had visual symptoms of PSS infection.

**Table 4:** The comparison of non-infected (non-symptomatic) compared to infected soybean seed as it influenced seed amino acids content. The field experiment was conducted in 2019 at the Delta Research and Extension Center in Stoneville, MS.

Measurement	Non-Infected ^x^	Infected ^y^	LSD	Pr > F
	(%)			
Valine	2.2 ^b^	2.4 ^a^	0.1	0.0335
Methionine	0.6 ^a^	0.6 ^a^	-	0.5536
Isoleucine	1.9 ^a^	1.9 ^a^	-	0.4833
Leucine	2.9 ^a^	2.9 ^a^	-	0.8864
Tyrosine	1.6 ^a^	1.6 ^a^	-	0.2244
Phenylalanine	1.8 ^b^	1.9 ^a^	0.1	0.0406
Lysine	2.7 ^a^	2.7 ^a^	-	0.6616
Histidine	0.7 ^a^	0.9 ^a^	-	0.1056
Arginine	2.6 ^b^	2.7 ^a^	0.1	0.0027

Means within a row followed by different letters are significantly different at *p* ≤ 0.05. ^x^ non-symptomatic seed showed no sign of purple seed stain (PSS); ^y^ infected seed had visual symptoms of PSS infection.

**Table 5:** The comparison of non-symptomatic compared to infected soybean seed as it influenced seed fatty acids content. The field experiment was conducted in 2019 at the Delta Research and Extension Center in Stoneville, MS.

Measurement	Non-Infected ^x^	Infected ^y^	LSD	Pr > F
	(%)			
Linoleic Acid	54.2 ^a^	53.3 ^a^	-	0.2845
Stearic Acid	4.5 ^a^	4.5 ^a^	-	0.8477
Palmitic Acid	9.9 ^a^	10.7 ^a^	-	0.1084
Oleic Acid	18.8 ^a^	18.9 ^a^	-	0.9070
Linolenic Acid	10.1 ^a^	10.1 ^a^	-	0.9225

Means within a row followed by different letters are significantly different at *p* ≤ 0.05. ^x^ non-symptomatic seed showed no sign of purple seed stain (PSS); ^y^ infected seed had visual symptoms of PSS infection.
